# Study on the Effect of Demulsification Speed of Emulsified Asphalt based on Surface Characteristics of Aggregates

**DOI:** 10.3390/ma11091488

**Published:** 2018-08-21

**Authors:** Fanlong Tang, Guangji Xu, Tao Ma, Lingyun Kong

**Affiliations:** 1 School of Transportation, Southeast University, Nanjing, Jiangsu 210096, China; 230179227@seu.edu.cn (F.T.); guangji_xu@seu.edu.cn (G.X.); 2 School of Civil Engineering & Architecture, Chongqing Jiaotong University, Chongqing 400074, China; klyyqr2002@163.com

**Keywords:** limestone aggregates, emulsified asphalt, demulsification speed, surface energy, specific surface area

## Abstract

Aggregate is an indispensable raw material for emulsified asphalt construction. For the purpose of explaining the influence of aggregate characteristics on the demulsification speed of emulsified asphalt, the surface energy and specific surface area (SSA) characteristics of aggregates were calculated based on the capillary rise method and the BET (Brunauer-Emmett-Teller) adsorption test. Afterwards, the effect of the surface energy and specific surface area of the aggregate on the emulsified asphalt demulsification speed was systematically studied by using ultraviolet spectroscopy as well as the orthogonal test. Experimental results indicate that the specific surface energy parameter of the aggregate is certainly related to the particle size of the aggregate. That is, the surface free energy of the unit system is proportional to the surface area A and the density of the interface unit. The specific surface area parameter of aggregates increases with the decrease of particle size, when the particle size is reduced to 600 mesh, the specific surface area parameters of the three aggregates selected in this paper tend to be consistent. Orthogonal experimental analysis demonstrates that the surface energy and specific surface area have an impact on the emulsion breaking speed and they are proven to be positively correlated. Meanwhile, in the case of small particle sizes, there is no statistically significant correlation between the physical properties of aggregates and the demulsification speed of emulsified asphalt, and the physical property of aggregates is not the main factor that affects the demulsification speed of the emulsified asphalt. On the contrary, the material properties of the aggregate, such as acid-base property and chargeability, are the dominant factors.

## 1. Introduction

With the advent of the world’s energy crisis and the enhancement of human consciousness of nature, emulsified asphalt, famous for its clean and eco-friendly properties, presents a prospect for large-scale use, and shows an increasing tendency of usage year-by-year [1,2,3].

By using this kind of asphalt emulsion to build a road, no heating is needed at room temperature to spray, to pour into or mix, or to pave the surface or base layer of various structural pavements. Emulsified asphalt can be used in normal temperature construction and has the advantages of saving energy, facilitating construction, saving asphalt, and protecting the environment [4,5,6]. In particular, the performance of emulsified asphalt modified with polymer is better than that without. It is generally expected that the asphalt modification can be used to help asphalt material to resist deformation at high temperature, and to resist cracking at low temperature. Thus, high-temperature rutting and low temperature cracking can be alleviated. Besides, it can not only greatly improve the interlayer combination of asphalt pavement, but also reduce the occurrence of various diseases in its service period. This provides great economic and social benefits in the maintenance of the road, especially for high grade pavement [7,8].

Emulsified asphalt property directly affects the production, storage, construction, and long-term serviceability of emulsified asphalt. During the production process, asphalt should be easily emulsified, and the emulsified asphalt has certain storage stability. However, in the construction process, it is hoped that the emulsified asphalt has an appropriate demulsification time, and it should fully meet the requirements of construction and to open traffic [9]. At the same time, emulsified asphalt should have good adhesion with aggregate, so that asphalt mixture has a strong ability to resist water damage [10,11]. Obviously, a controllable emulsified asphalt demulsification is extremely important for the performance of emulsified asphalt. Nevertheless, in practical projects, due to poor knowledge of action mechanisms between emulsifier and aggregate which lead to the un-controlled breaking conditions of asphalt emulsion, and the instable long-term performance seriously restricts the large-scale use of energy-saving and environmental-friendly emulsified asphalt mixture. Based on this, a lot of previous studies on the controllable of demusification have been carried out, mainly on the emulsifier, the type and amount of additives, the emulsified asphalt preparation conditions and so on. MARCHAL et al. found that the amount of emulsifier that is not combined with asphalt or adsorbed on the surface of the aggregate, greatly affects the demulsification speed of the asphalt emulsion [12,13]. According to the analysis of Xie et al., the particle size, density, and continuous phase viscosity have an essential influence on the demulsification speed of the emulsified asphalt [14]. GORMAN et al. explained that the most important factors affecting the demulsification process of emulsified asphalt are the performance of emulsified asphalt and the surface properties of the aggregate [15]. HAGEN et al. concluded that the surface properties of the aggregate have great influence on the demulsification speed and adhesion of emulsified asphalt [16]. ADDERSORI [17] and CASTILLO [18] think the desorption of emulsifier on the asphalt surface and the adsorption on the aggregate surface affect the demulsification of emulsified asphalt. SHELL et al. showed that the increase of temperature can accelerate the breaking speed of the emulsified asphalt [19]. 

Aggregate is an important raw material and it directly affects the controllability of emulsified asphalt demulsification, which is highly related to the efficiency of the emulsified asphalt construction. However, the research on the controllability of emulsified asphalt demulsification speed based on an aggregate factor, especially the surface characteristics of the aggregate, are rarely reported. In this paper, based on the surface properties of aggregates, the surface energy and specific surface area parameters of the aggregates are qualitatively characterized first. Additionally, the influence of the physical surface characteristics of aggregates on the demulsification speed of emulsified asphalt was systematically evaluated by means of UV spectroscopy and an orthogonal test. The objective of this study intends to give an explanation for the impact of emulsion asphalt breaking speed from the view of the surface characteristics of the aggregate, and it provides a theoretical reference for the aggregate factor, to determine the controllable demulsification of emulsified asphalt.

## 2. Materials and Methods

### 2.1. Materials

#### 2.1.1. Emulsified Asphalt Selection

**Figure 1:**
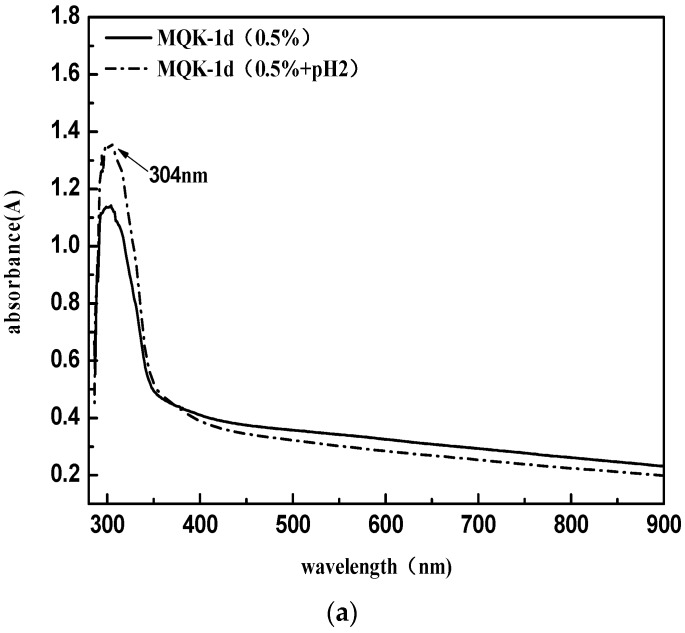

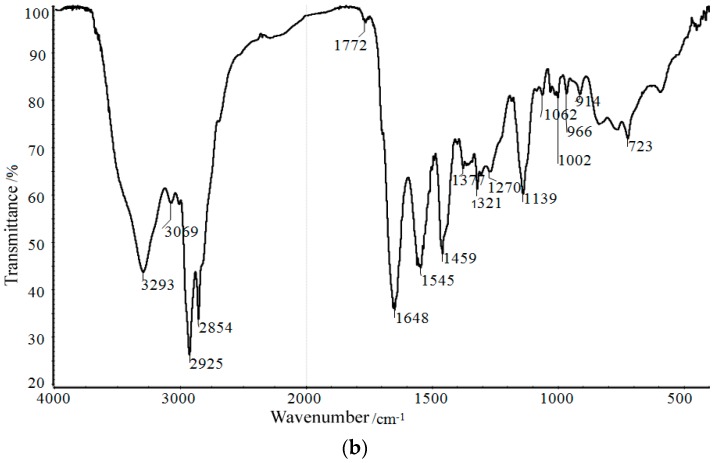
MQK-1D ultraviolet spectrum (**a**) and Infrared Spectrogram (**b**).

In this paper, the KunLun AH-70 asphalt was chosen as the matrix asphalt, and the corresponding cationic emulsified asphalt was used [20]. A slow-cracking and fast-setting type emulsifier named MQK-1D from MeadWestvaco Corporation in the United States was selected. It mainly consists of amino amide compounds generated from fatty acid and polyamine condensation [21] (under the ultraviolet spectrum, the absorption peak position is λ_max_ = 304 nm). Emulsified asphalt properties are presented in in Table 1. MQK-1D Infrared Spectrogram and UV spectrum are shown in Figure 1.

Since the emulsified asphalt in this research is cationic emulsified asphalt, blue or red shifts of the peaks of the emulsifier may occur in the ultraviolet spectrum under the environment of pH = 2. Thus, the comparison data MQK-1D (0.5% + pH2) were added. Test results suggest that the pH value has no significant effect on the position of the emulsifier peak, and thus it can be considered λ_max_ = 304 nm.

#### 2.1.2. Aggregate Selection

Representative domestic road aggregates were selected, followed by limestone, granite, basalt, and quartzite. Particle size distribution is 200 mesh, 300 mesh, 400 mesh, 500 mesh (mesh which is used to indicate the particle size of particles that can pass through the mesh. The higher the mesh number, the smaller the particle size [24]). Then, the four aggregates were sampled by a quartering method [25], and the crush value test, abrasion value test, adhesion test, and apparent density test were performed. The basic performance indexes are shown in Table 2.

According to Table 2, it can be seen that the basic properties of the above four aggregates were entirely able to meet the requirements of JTG E42-2005 of the Highway Engineering Aggregate Test Procedures and they could be used as tests.

### 2.2. Characterization of Emulsion Asphalt Demulsification Speed

Emulsified asphalt is a black, viscous emulsion at room temperature. When the external conditions change (mechanical agitation, aggregate mixing, demulsifiers), demulsification occurs. However, in the process of demulsification, it is difficult to find the essential difference in the appearance, and it is impossible to directly evaluate the emulsion demulsification speed. Nevertheless, the nature of the demulsification process of emulsified asphalt lies in the separation of oil and water phases. In the centrifugal field outside, oil in water equilibrium system is broken up. The asphalt droplets, which are in a dispersed phase, rapidly aggregate and sink. With the continuous development of emulsified asphalt demulsification, the concentration of emulsifier in the supernatant is continuously increased until the equilibrium of emulsion breaking is reached. Based on this, this study used the UV spectroscopy analysis to characterize the breaking speed of emulsified asphalt by examining the change of emulsifier concentration in the supernatant.

Ultraviolet spectroscopy is based on UV-visible spectrophotometry, which is generally considered to be 200–800 nm, and is a spectral analysis method based on the molecules of different substances [26,27]. According to the Lambert-Beer law, as a beam of parallel monochromatic light passes through an ideal solution of a single homogeneous, the absorbance and concentration of the solution always exhibits a direct positive proportional relationship.

The procedure can be briefly described as follows: First, 40 g of an equal amount of emulsified asphalt was centrifugated (centrifugation temperature 20 °C) for different times. After this, 1 mL supernatant with pipette was diluted with 50 mL distilled water for UV measurement. In order to obtain the proper simulation conditions for the demulsification process, the process was repeated three times first [28]. From the figure below, simulation process 1 used a centrifugal speed of 3000 rpm; the experimental conditions performed in process 2 were invariable, while a second supernatant centrifugation was required. The detailed method of the second centrifugation is that the obtained solution was placed in a centrifuge tube and subjected to high-speed centrifugation again. Because of the difference in density, the asphalt particles were adsorbed onto the tube wall by the action of a high-speed centrifugal field, so that the elimination of pitch particles suspended in solution in the case of a constant concentration of emulsifier was ensured; simulation process 3 was exactly the same as simulation process 2 except that the centrifugal speed was adjusted to 1500 rpm.

**Figure 2:**
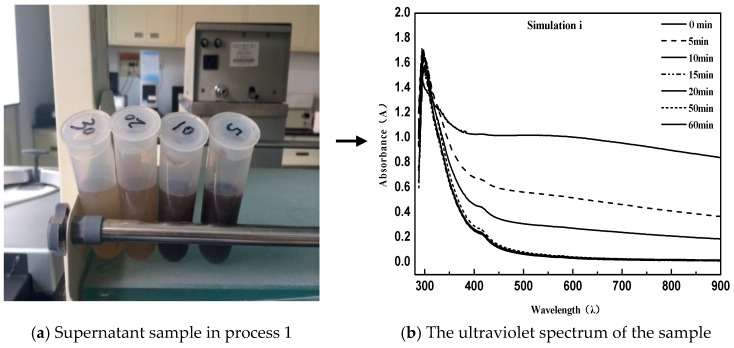
Direct sampling simulation for process 1.

**Figure 3:**
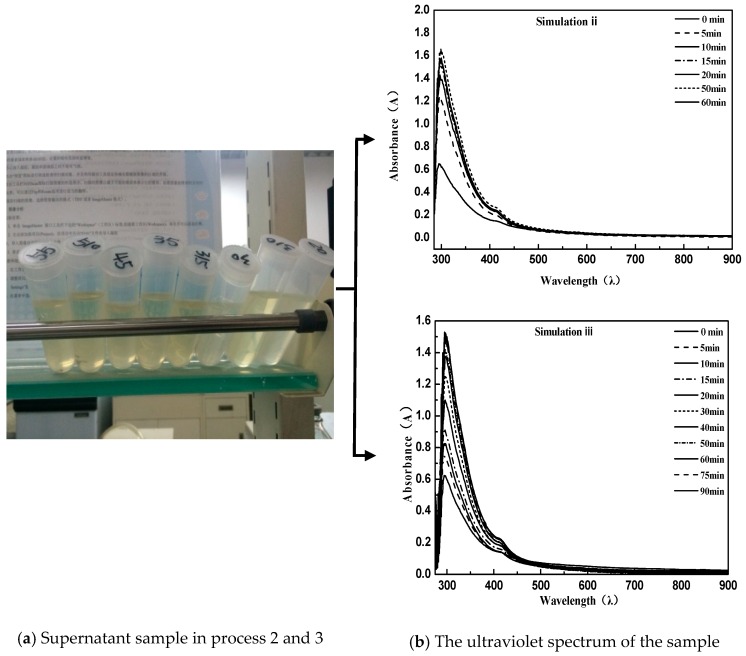
Using secondary centrifugal sampling for simulation process 2 and simulation process 3.

As can be seen from the above Figure 2 and Figure 3, the directly sampled supernatant was turbid, and the solution contained a large amount of suspended asphalt particles. This phenomenon had a great deviation from the experimental results. Simultaneously, it led to the disruption of the UV spectrum measurement and the inability to characterize the emulsion demulsification process. Instead, the treated supernatant contained very few suspended asphalt particles with a clear and transparent solution; meanwhile, the obtained UV spectrum data were consistent with the emulsified asphalt emulsion breaking rules.

Comparing simulation Process 2 and Simulation Process 3, the emulsified asphalt broke so fast that the demulsification procedure was complete at 15 min at a speed of 3000 rpm. However, a uniform intercept UV spectrum curve appeared in the emulsified asphalt demulsification process when the speed is reduced to 1500 rpm. This indicates that the simulated demulsification process of emulsified asphalt was met under the conditions of a moderate demulsification speed of 1500 rpm.

## 3. Aggregate Characteristics

### 3.1. Aggregate Surface Energy Measurement

**Figure 4:**
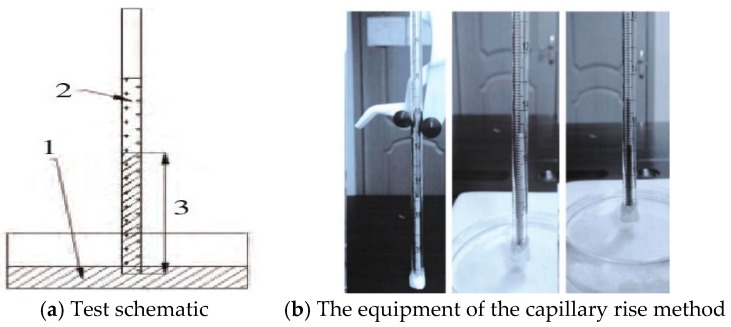
Schematic diagram of the capillary rise method.

The approach applied for surface energy trial in our research is known as the capillary rise method [29,30]. The basic principle of this method is that tiny capillary channels will form in the powder gap between solid powders. Through capillary action, liquids can spontaneously penetrate into the powder column in the glass tube by measuring a liquid with known surface tension. While the liquid rises to a certain height and takes time, the contact angle information of the liquid to the powder can be obtained. The principle of experimental is shown in Figure 4.

Experiment conditions are described as follows: The environment temperature was 25 °C, the reagent was weighed uniformly to 0.50 g using an analytical balance, the capillary was shaken for 5min after injecting the ore powder, and 0.20 mm thick filter paper was stuck onto the bottom of the capillary. The capillary was a pipette with a length of 20 cm and an internal diameter of 2.7 mm. The surface energy parameters of pentane and the immersion liquid used in the experiment are shown in Table 3.

**Figure 5:**
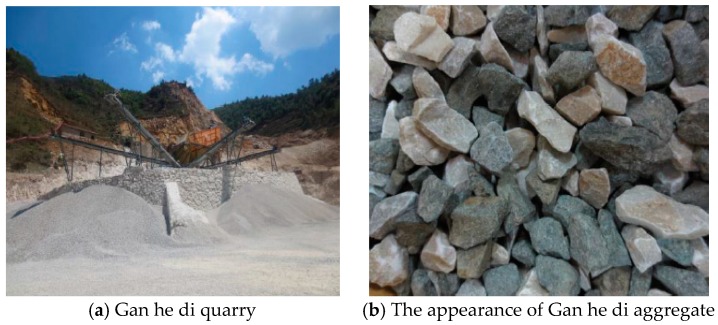
Gan he di quarry (**a**) and stone appearance (**b**).

**Figure 6:**
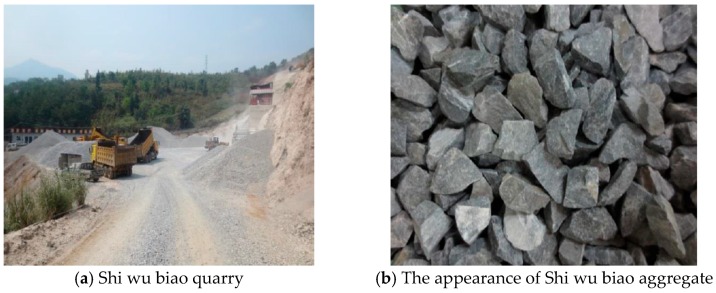
Shi wu biao quarry (**a**) and stone appearance (**b**).

**Figure 7:**
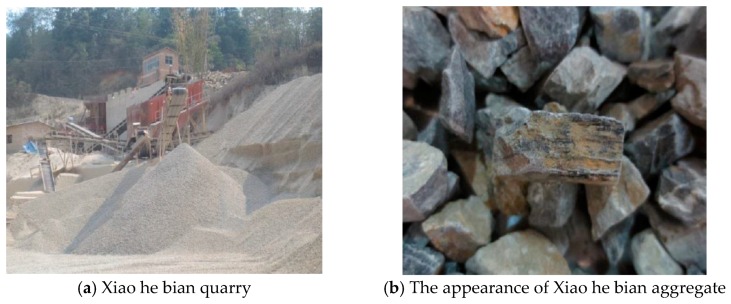
Xiao he bian quarry (**a**) and stone appearance (**b**).

The following experiments are the surface energy measurements of limestone. Three types of limestone aggregates with different appearances and different sampling points are from Gan he di (Figure 5), Shi wu biao (Figure 6), and Xiao he bian quarries (Figure 7).

In order to reduce the number of experiments and possible chemical interference in the study of physical properties, this test was only performed for limestone aggregates (the effective radius of this experiment was calibrated to use a low surface energy liquid pentane with a contact angle *θ* close to zero).

**Figure 8:**
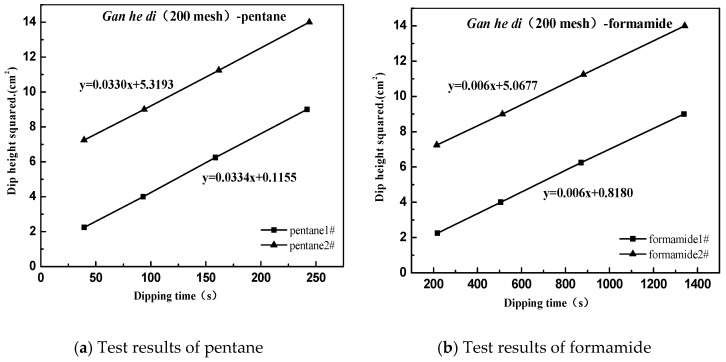

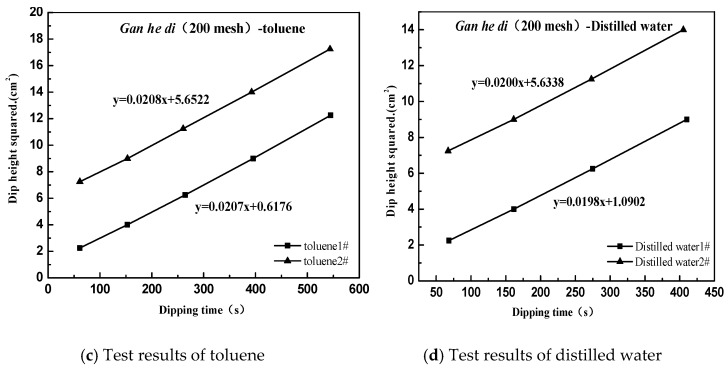
Surface energy test of aggregate. (**a**) pentane; (**b**) formamide; (**c**) toluene; (**d**) distilled water.

The following case uses Gan he di (200 mesh) as an example, and the result as shown in Figure 8.

According to the Washburn immersion equation [31], the contact angles for different types of ore powder under different test liquid conditions are calculated as follows:

(1) $$h^{2}/t=(\gamma_{1}R_{eff}\cos\theta )/2\eta$$

In the formula, *h* is the liquid rising height, cm; *η* is the viscosity of the liquid, mN·m^−2^·s^−1^; *R_eff_* is the effective radius of the capillary, μm; *θ* is the contact angle between liquid and solid; *t* is the dipping time, *s*; *γ_l_* is the free surface energy of the liquid, mJ·m^−1^.

Then according to the equation of Young, the formula is as follows:

(2) $$\gamma_{1}(1+\cos\theta )=2\sqrt{\gamma_{sv}^{d}\gamma_{lv}^{d}}+\sqrt{\gamma_{sv}^{p}\gamma_{lv}^{p}}$$

Given that:

(3) $$y=\frac{1}{2}\left(1+\cos\theta \right)\frac{\gamma_{l}}{\sqrt{\gamma_{l}^{d}}},x=\sqrt{\frac{\gamma_{l}^{p}}{\gamma_{l}^{d}}}$$

Based on the above test results, a relationship diagram was established between *y* and *x*, where the square of the slope is the polar force component of the measured interface, and the square of the intercept is the dispersion force component of the measured interface. The dispersion force component of Gan he di (200 mesh) is calculated to be 19.85 mJ·m^−2^, the polar force component is 24.13 mJ·m^−2^, and the final surface energy is 43.98 mJ·m^−2^.

**Figure 9:**
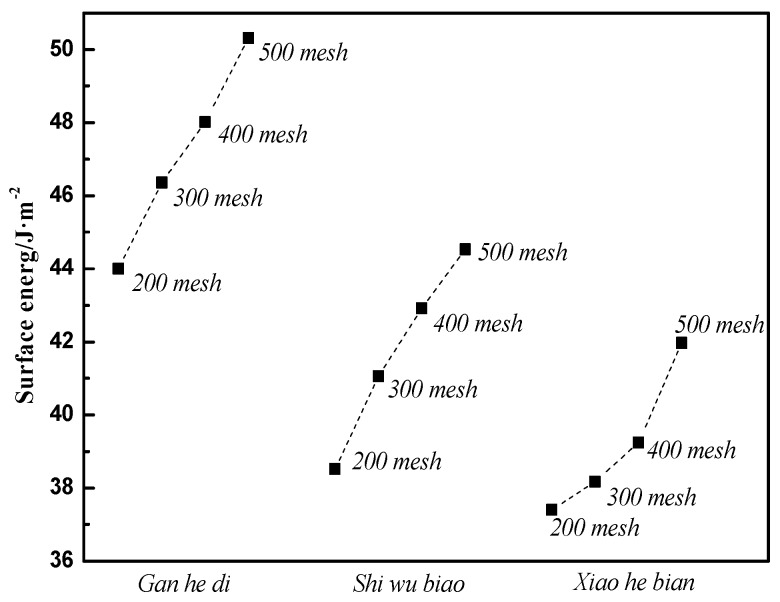
Surface energy of three kinds of aggregates.

Similarly, experiments and calculations were carried out on the Gan he di (300 mesh, 400 mesh, 500 mesh), as well as on the Xiao he bian and Shi wu biao. Finally, the surface energy parameters of three kinds of limestone aggregates with different particle sizes are shown in Figure 9.

### 3.2. Determination of Specific Surface Area of Aggregate

Based on the basic principle of BET adsorption, the research measured specific surface area for three limestone with particle sizes of 200 mesh, 300 mesh, 400 mesh, 500mesh, and 600mesh, respectively by using ASAP2020M+C physical adsorption instrument (made in Shanghai China by the corp. of Micromeritics).

**Figure 10:**
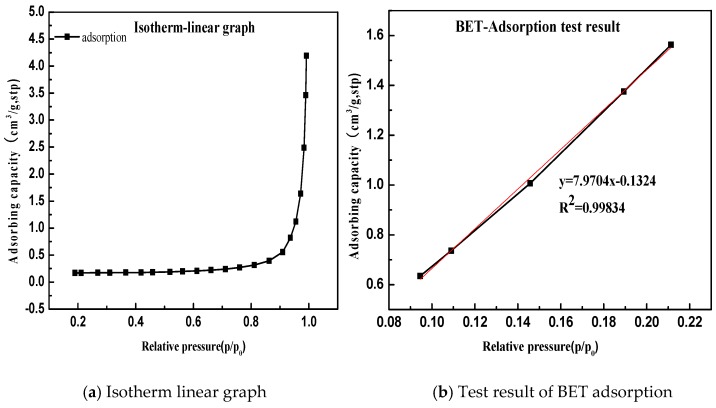
Determination of specific surface area of limestone. (**a**) Isotherm linear graph; (**b**) BET adsorption test result.

Take Gan he di as an example, the specific surface area result is shown in Figure 10.

According to the BET equation, the Gan he di (200 mesh) has a specific surface area of 0.339 m^2^/g. The test and calculation methods for the remaining samples are similar, and the calculation results are summarized in Table 4.

**Figure 11:**
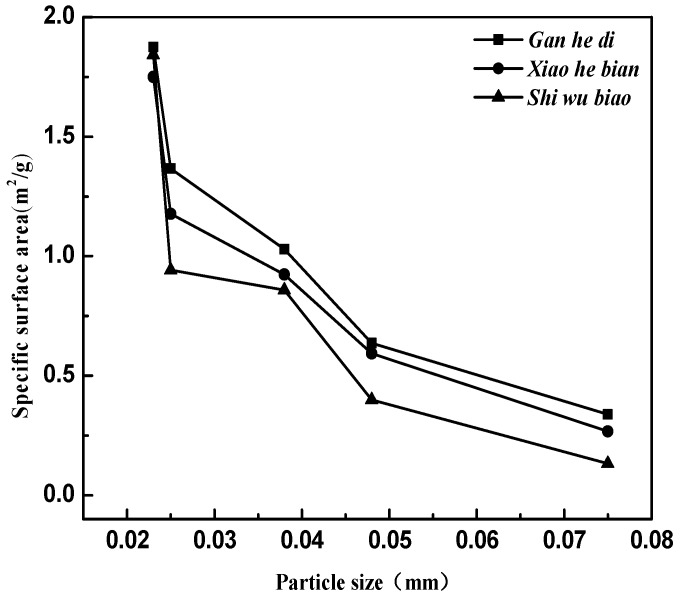
The specific surface area of three kinds of aggregates with different particle sizes.

The relationship between the specific surface area of the limestone aggregates and the particle size is established, as shown in Figure 11.

As shown in Figure 11, the specific surface area of the three aggregates decreases with particle size increasing. For the three limestone aggregates selected in the test, the specific surface area of the Gan he di is the largest, and the specific surface area of Shi wu biao is the smallest as the grain size exceeds 600 mesh; While the particle size is reduced to 600 mesh, the specific surface area of the three types of aggregates remains basically consistent.

## 4. Results and Discussion

### 4.1. Orthogonal Design

It is well known that the specific surface area and surface energy are directly related to the material properties of the aggregate and the particle size of the aggregate [32,33]. On the other hand, the demulsification speed of the emulsified asphalt also has an objective relationship with the selection of the mixing ratio and the demulsification time node. Therefore, the experiment adopts orthogonal test design to select three different particle size limestone aggregates with different performance and composition and the effects of surface energy and specific surface area of the aggregate on the emulsion breaking speed of emulsified asphalt were investigated from the time factor, material factor, mixing ratio factor and particle size factor. The experimental method is the ultraviolet spectrometry obtained in Section 2.2. The factor level table as shown in Table 5.

Select the L9 (3^4^) orthogonal table, seven times for each test condition, average the results, and fill in the data in Table 6.

**Figure 12:**
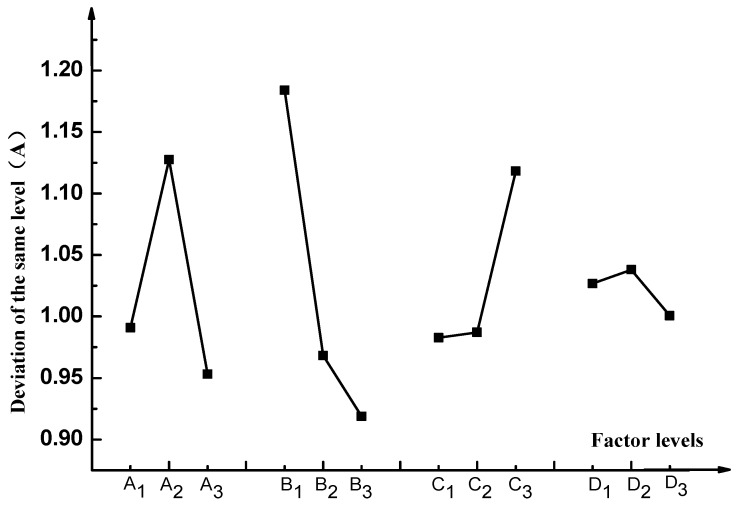
Factors and indexes.

Figure 12 shows the relationship between indicators and factors.

As can be seen from Figure 12:

(1) The factor A (time) and factor B (material) had large deviations, therefore, this factor was the main factor influencing the breaking speed of emulsified asphalt. For factor C (mixing ratio), the deviation is barely noticeable difference at level 1 (5%) and level 2 (10%), while the deviation was obvious when level 3 (20%) was reached, which reveals the effect of emulsified asphalt on the rate of demulsification is not obvious when the mixing is relatively low. But when the mixing ratio is increased to a certain value, the demulsification speed will change significantly.

(2) From the Figure 12, the deviation of the factor D (particle size) was the smallest, which indicates that the particle size factor had a lower impact on the breaking speed of the emulsified asphalt. The reason for the analysis may be due to the small difference in the particle size of the aggregates selected in this experiment.

Next, factors B (material factor) and D (particle size factor) were used to analyze the influence of surface energy and the specific surface area of the aggregate on the breaking speed of the emulsified asphalt.

### 4.2. Effect of Aggregate Surface Energy on Demulsification Rate of Emulsified Asphalt

In order to study the influence of the surface energy parameters of aggregate on the breaking speed of emulsified asphalt, correlation analysis was carried out according to Figure 12 from the factors of material and particle size.

**Figure 13:**
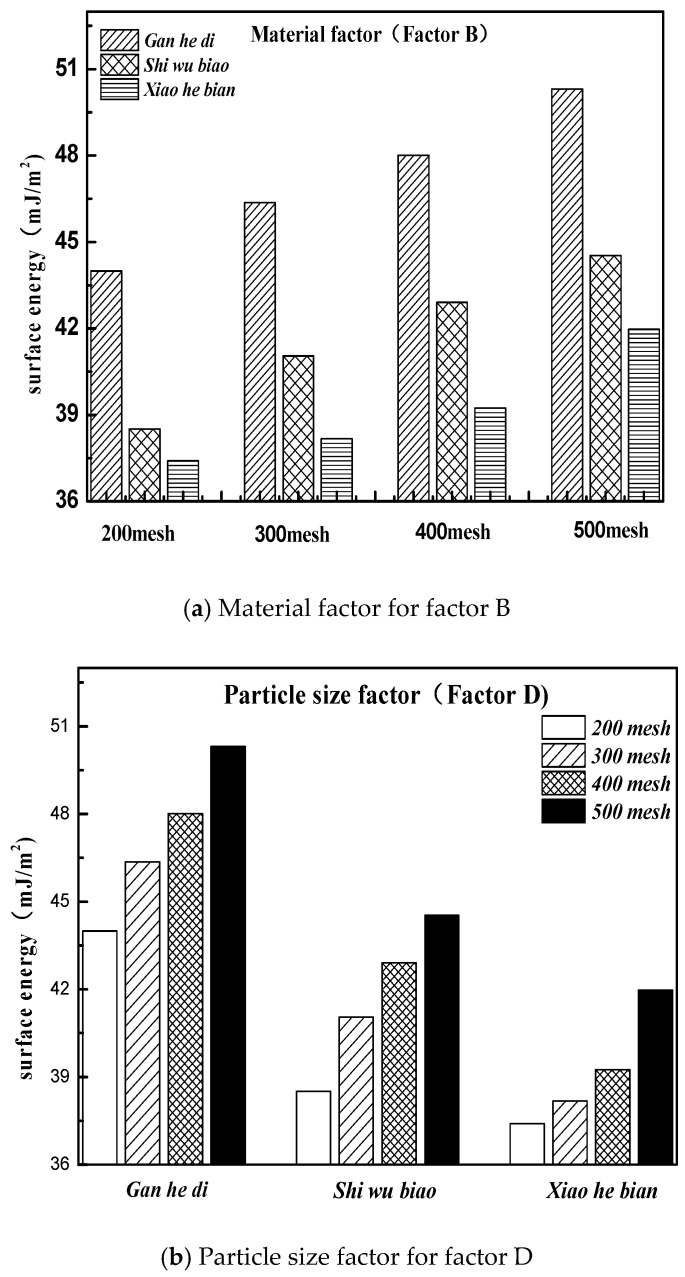
Material and particle size factors under the three kinds of aggregate surface energy parameters. (**a**)Material factor for factor B; (**b**) Particle size factor for factor D.

According to the design conditions of the orthogonal experiment in the previous section, it can be seen that level 1 of factor B was Gan he di, level 2 was Shi wu biao, and level 3 was Xiao he bian. The surface energy parameters of three different aggregates were compared, as shown in Figure 13.

Figure 13 shows that the surface energy parameters of aggregates are related to the material itself and the particle size. For the three aggregates studied in this experiment, the surface energy parameters of the Gan he di aggregates are the largest, followed by the Shi wu biao and the minor ones Xiao he bian. In combination with Figure 12, under the material factor, level 1 (Gan he di) has the fastest demulsification rate, while level 3 (Xiao he bian) has the slowest rate of demulsification. Under the particle size factor, level 2 (500mesh) has the fastest demulsification rate and level 3 (200mesh) has the slowest rate of demulsification, which means that the greater the surface energy of the aggregate, the faster the breaking speed of emulsified asphalt. However, compared with the material factor, the influence of the particle size factor on the demulsification speed was less biased, which means that for the material factor, the surface energy parameter was not the only factor affecting the demulsification speed of emulsified asphalt. Combining with particle size analysis, it can be inferred that the influence of this unknown factor on the emulsion demulsification speed of emulsified asphalt should be larger than the surface energy parameters. The analysis of the unknown factors here is related to the structure, porosity, acid-base property of the material.

### 4.3. Influence of Specific Surface Area of Aggregate on Demulsification Speed of Emulsified Asphalt

**Figure 14:**
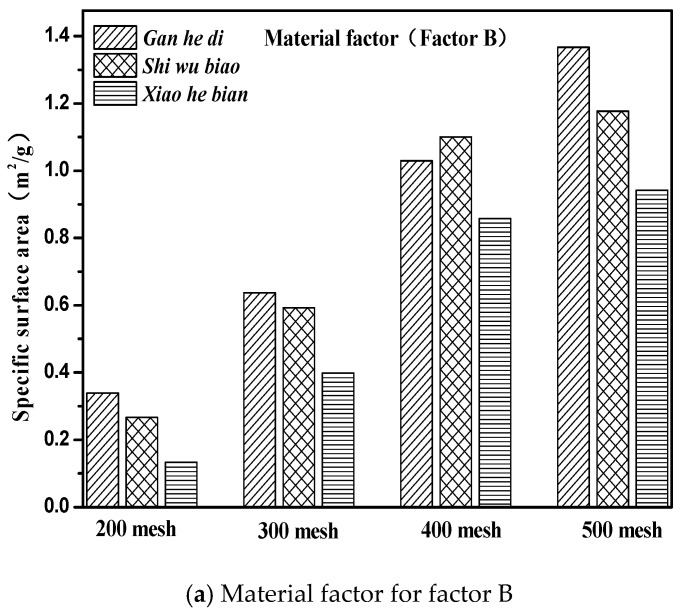

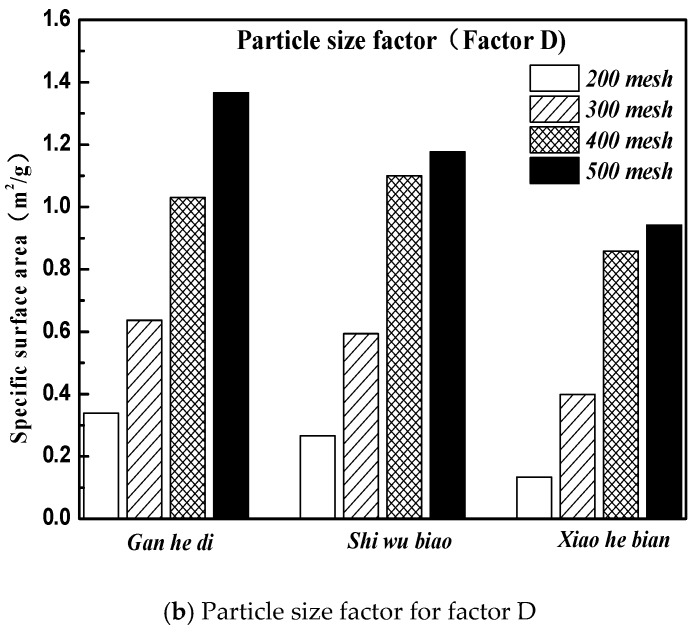
Material and size factors under three kinds of aggregate surface parameters. (**a**) Material factor for factor B; (**b**) Particle size factor for factor D.

The specific surface area parameters of the three aggregates of different particle sizes studied in this paper were demonstrated in Section 3.2. The specific surface area parameters of the aggregates are compared with the correlation graphs of the factors and indicators in Figure 12, and results are shown in Figure 14.

It can be seen from Figure 14 that the specific surface area parameters of aggregates decreased with the increase of particle size, and the specific surface area parameters of different aggregates were not the same. The specific situation was 500 mesh > 400 mesh > 300 mesh > 200 mesh; Gan he di > Shi wu biao > Xiao he bian. The surface energy parameter of Shi wu biao, with a particle size of 400 mesh, was slightly larger than that of Gan he di, which may have been due to the random error generated by the experiment). In order to study the relationship between the specific surface area parameters of aggregates and the emulsion breaking speed of emulsified asphalt, the grey correlation was used for analysis. The results are shown in Table 7.

According to Table 7, Figure 13 and Figure 14, the specific surface area of the aggregate was positively related to the emulsion breaking speed of the emulsified asphalt; that is, the larger the specific surface area is, the faster the emulsified asphalt breaks. From the gray correlation analysis in Table 7, it can be seen that the relationship between the specific surface area parameters of the aggregate and the breaking speed of emulsified was relatively low, indicating that the specific surface area parameter of the aggregate was not the dominant factor affecting the breaking speed of emulsified asphalt.

## 5. Conclusions

(1) The capillary rise method was used to determine the surface energy of aggregates, and results suggest that the specific surface energy parameters of aggregates are related to the particle size of aggregates. The larger the particle size, the smaller the surface energy, and vice versa. Besides, the surface free energy of the unit system is always proportional to the surface area A, as well as the density of the interface unit. The BET adsorption test illustrates the specific surface area of aggregates increases with the decrease of particle size; however, the specific surface area parameters of the three aggregates selected in this paper tend to be consistent when the particle size is reduced to 600 mesh.

(2) The influence of the surface energy and specific surface area of the aggregate on the breaking speed of emulsified asphalt was evaluated by UV spectroscopy. It was concluded that the surface energy and the specific surface area are exactly related to the emulsion breaking speed of the emulsified asphalt, and they are positively correlated. The larger the surface energy and the specific surface area of the aggregate, the faster the emulsion breaking speed of the emulsified asphalt.

(3) Gray correlation analysis shows that the correlation between the physical properties (surface energy, specific surface area, particle size) of the aggregate and the emulsified asphalt demulsification is relatively low when the aggregate size is small (mineral powder), and it indicates that physical microscopic characteristics are not the main factor that affect the emulsion breaking speed. Instead, the material properties (excluding physical properties) of the aggregate itself, such as acid-base properties and chargeability, are the dominant factors affecting the emulsified asphalt demulsification.

(4) The effect of the characteristics of aggregates on the emulsion breaking speed of emulsified asphalt, in this article, is restricted to small particle size fine aggregates (as well as mineral powder). The effects of the relevant characteristics of coarse aggregates on the demulsification speed of emulsified asphalts are currently unable to be determined. Therefore, it has to be admitted that conclusions have certain limitations and further research is needed. Besides, for the coarse aggregates, a significant change, as revealed in the particle size, further study on the relationship between the aggregates and demulsification speed is necessary for.

## Figures and Tables

**Table 1:** Emulsified asphalt performance properties [22,23].

Item		Unit	Required Value	Experimental Value
Emulsifier dosage		wt %	-	2
Appearance		-	Ecru liquid, uniformity, without mechanical impurities	qualified
Particle charge		-		positive
Engler viscosity (25 °C)		-	5–30	3.2
Plus sieve surplus (1.18 mm)		%	≤0.1	0
Evaporated residue	Leftover content	%	≥60	60.86
	Penetration (25 °C)	0.1 mm	40–120	57
	Ductility (15 °C)	cm	≥50	69
	Softening point	°C	-	47.9
Cement hybridism		%	<1.0	0.45
Storage stability	1 day	%	≤1.0	5.9
	5 day	%	≤5.0	10.85

**Table 2:** The basic performance of the aggregate.

Technical Indicators	Crushing Value/%	Los Angeles Wear Value/%	Adhesion Grade With Asphalt	Apparent Density/g/cm^3^
Limestone	18.2	15.8	5	2.700
Basalt	9.7	10.3	3	2.707
Granite	15.3	14.1	3	2.677
Quartzite	16.3	12.6	3	2.625
Requirements	≤28	≤30		

**Table 3:** Surface energy parameters of an immersed liquid.

Reagent	Surface Free Energy/mJ·m^−2^	Dispersion Component	Polar Component	Viscosity/mPa·s
Distilled water	72.80	21.80	51.00	0.890
Formamide	58.00	39.00	19.00	3.343
Toluene	27.70	27.70	0	0.560
N,N Dimethylformamide	37.30	32.42	4.88	0.796
Diiodomethane	50.80	50.80	0	1.220
Pentane	15.49	15.49	0	0.224

**Table 4:** Summary of SSA calculation (m^2^/g).

Specimen	200 mesh	300 mesh	400 mesh	500 mesh	600 mesh
*Gan he di*	0.3388	0.6369	1.0296	1.3669	1.8751
*Xiao he bian*	0.1334	0.3987	0.8579	0.9422	1.8429
*Shi wu biao*	0.2667	0.5935	1.1000	1.1770	1.7493

**Table 5:** Factor levels table.

	Factor	A Time (min)	B Material	C Mixing Ratio (%)	D Particle Size (mesh)
Level					
1		25	*Gan he di*	5	300
2		30	*Shi wu biao*	10	500
3		20	*Xiao he bian*	20	200

Note: In order to minimize the random errors that occur in the test, the ordering of the horizontal numbers here adopts random sorting.

**Table 6:** Test plan and test result analysis.

TEST PLAN						Test Results
Index	**Factor Test Number**	**A**	**B**	**C**	**D**	**Absorbance(A)**
		**1**	**2**	**3**	**4**	
	1	1(25 min)	1(*Gan he di*)	1(5%)	1(300 mesh)	1.0961
	2	1	2(*Shi wu biao*)	2(10%)	2(500 mesh)	1.2338
	3	1	3(*Xiao he bian*)	3(20%)	3(200 mesh)	1.2219
	4	2(30 min)	1	2	3	0.9129
	5	2	2	3	1	1.1697
	6	2	3	1	2	0.8732
	7	3(20 min)	1	3	2	0.9632
	8	3	2	1	3	0.9792
	9	3	3	2	1	0.8243
Emulsified asphalt Demulsification speed	$K_{1}$	2.9722	3.5518	2.9485	3.0801	T = $K_{1}+K_{2}+K_{3}$
	$K_{2}$	3.3827	2.9058	2.9610	3.1140	
	$K_{3}$	2.8594	2.7567	3.3548	3.0022	
	${\bar{K}}_{1}(K_{1}/3)$	0.990733	1.1838667	0.98283	1.0267	
	${\bar{K}}_{2}(K_{2}/3)$	1.127567	0.9683	0.987	1.038	
	${\bar{K}}_{3}(K_{3}/3)$	0.953133	0.9189	1.11827	1.0007	
	*R*	0.174437	0.264967	0.13544	0.0373	

**Table 7:** The specific surface area of the aggregate and the demulsification of the emulsified asphalt speed of grey correlation analysis.

	Material Factors				Particle Size Factor		
Gray correlation coefficient	200 mesh	300 mesh	400 mesh	500 mesh	*Gan he di*	*Shi wu biao*	*Xiao he bian*
	0.3885	0.4490	0.7430	0.7088	0.5760	0.5500	0.4574
